# Impedance Aggregometry Reveals Increased Platelet Aggregation during Liver Transplantation

**DOI:** 10.3390/jcm8111803

**Published:** 2019-10-27

**Authors:** Mohamed Soliman, Matthias Hartmann

**Affiliations:** Klinik für Anästhesiologie und Intensivmedizin, Universitätsklinikum Essen, Universität Duisburg-Essen, 45122 Essen, Germany; mohamed.soliman@stud.uni-due.de

**Keywords:** liver transplantation, platelet aggregation, impedance aggregometry, Multiplate™, von Willebrand factor, ristocetin

## Abstract

In patients presenting for liver transplantation, increased platelet aggregation as well as thrombocytopenia have been demonstrated, but bedside assays have not been investigated. We compared platelet aggregation in liver transplantation patients and control surgical patients using impedance aggregometry. We hypothesized that platelet activity is not altered during liver transplantation. After the allowance of the ethics committee, platelet aggregation was determined using impedance aggregometry with the activators ristocetin, adenosine diphosphate (ADP), arachidonic acid, collagen, and thrombin receptor-activating peptide (TRAP) in liver transplantation patients at four time points (start of surgery, anhepatic phase, reperfusion, end of surgery) and in control surgical patients. Moreover, platelet count was determined using a Coulter counter. To compensate for the thrombocytopenia often present in patients presenting for liver transplantation, the ratio between impedance aggregometry finding and platelet count was used. For statistical evaluation, the *t*-test or the Mann–Whitney U-test were used, as appropriate. Platelet aggregation ratio showed a 3.1-fold increase in liver transplantation patients (*n* = 37) in comparison to control surgical patients (*n* = 10) when ristocetin was used as the activator (*p* = 0.001). Moreover, an approximately twofold increase of ADP-, arachidonic acid-, collagen-, and TRAP-induced platelet aggregation ratio was determined. Platelet aggregation normalized at the end of the transplantation procedure. Impedance aggregometry revealed a markedly increased platelet aggregation in some liver transplantation patients and might be suitable to guide platelet transfusion and antiplatelet therapy.

## 1. Introduction

In patients with end-stage liver failure presenting for liver transplantation, numerous changes in hemostasis and platelet function can be observed [1]. Liver failure leads to a reduced synthesis of coagulation factors as well as anticoagulant and fibrinolytic proteins in the liver. The result of the alterations is the so-called rebalanced hemostasis, with no bleeding tendency under physiological conditions but risk for both bleeding and thrombosis during transplantation [2,3].

Concerning primary hemostasis, thrombocytopenia, which is common due to reduced platelet production and consumption, may be counteracted by increased von Willebrand multimer levels, which are highly active activators of platelet aggregation [4,5,6]. The production of von Willebrand multimers in the endothelium may increase due to the fact that liver diseases are often linked to inflammation [4]. Moreover, the degradation and thus inactivation of highly active multimers by the cleaving enzyme ADAMTS13 synthesized in the liver may be reduced [7,8,9]. In end-stage liver disease, platelets are involved in liver inflammation, fibrosis, and even hepatocellular carcinoma development [10]. In liver transplantation patients, platelets can be trapped in the transplant, leading to the sinusoidal obstruction syndrome [11,12]. It is thus plausible that the monitoring of platelet function might be beneficial during liver transplantation. Unfortunately, platelet function testing is commonly performed by time-consuming Born aggregometry in specialized laboratories and, thus, monitoring is not available during the transplantation procedure. In contrast, whole-blood impedance aggregometry with the Multiplate™ analyzer is a new bedside method for the measurement of platelet function within a few minutes [13]. The method is capable to measure the aggregation of platelets on electrodes in whole-blood samples induced by adenosine diphosphate (ADP), collagen, arachidonic acid, thrombin receptor-activating peptide (TRAP), and ristocetin to monitor the effects of antiplatelet agents as well as von Willebrand factor activity. Therefore, the method might be useful for the judgement of platelet aggregability in patients during liver transplantation. In contrast to conventional Born aggregometry, impedance aggregometry samples are not corrected for platelet count, and the effect of thrombocytopenia in end-stage liver disease on the readings has to be addressed by the calculation of the ratio between impedance aggregometry findings and platelet count. It was the aim of the present study to investigate platelet function with the multiplate™ device during liver transplantation and in control patients and to evaluate the correction of impedance aggregation findings for differences in platelet count.

## 2. Materials and Methods

### 2.1. Patients

Data from 37 patients (33 adults, 4 children, 13 females, 24 males) with liver disease presenting for orthotopic liver transplantation and from 10 control surgical patients were retrospectively evaluated after allowance from the local ethics committee of the University Hospital Essen. The diseases leading to liver transplantation were hepatitis [14], alcohol consumption (*n* = 5), cholestatic liver disease (*n* = 4), nonalcoholic fatty liver disease (*n* = 3), biliary atresia (*n* = 3), autoimmune diseases (*n* = 2), and others including unknown causes (*n* = 6).

Liver transplantations were performed with organs from deceased donors. Surgery was performed with a vena cava replacement technique. None of the transplanted patients underwent a venovenous bypass.

### 2.2. Blood Sampling

At our institution, blood samples are drawn in a standardized manner from patients undergoing liver transplantation at 4 fixed time-points: skin incision, 10 min after the begin of the anhepatic phase, 10 min after reperfusion of the transplant, and at the end of surgery. Blood samples for the determination of platelet function with impedance aggregometry were collected in hirudin-containing tubes (Sarstedt, Germany). Platelet count was determined from blood sampled in EDTA-anticoagulated tubes (Sarstedt, Germany).

### 2.3. Impedance Aggregometry and Coulter Counter Measurements

For impedance aggregometry, the Multiplate™ device (Roche, Switzerland) was used. Platelets were activated with ristocetin, ADP, arachidonic acid, collagen, and TRAP. For the platelet function tests, hirudin-treated whole-blood samples were subjected to the Multiplate^®^ analysis according to the manufacturer’s recommendations. In short, a total of 300 μL saline and 300 μL heparinized whole-blood aliquots were added to the test cell. After three minutes of incubation at 37 °C, the samples were activated with 20 µl of the following activators: ristocetin, arachidonic acid, ADP, collagen, or TRAP, prepared according to the manufacturer´s recommendations. Platelet aggregation findings were assessed by the determination of the area under curve in arbitrary units (AU).

Platelet count was measured with a Coulter counter (KX-21N; Sysmex, Germany).

### 2.4. Calculations

In a recent experimental study, we demonstrated that platelet function readings measured with impedance aggregometry increase proportionally with platelet count. In 8 experiments, the correlation between these variables was 0.90 ± 0.07 (*p* < 0.0001). The ratio between platelet function reading and platelet count was shown to be independent of platelet count and allows to compare the platelet function of groups with different platelet counts (manuscript in preparation). In the present study, both platelet function readings obtained with impedance aggregometry and ratios are given.

### 2.5. Statistics

IBM SPSS™ statistics version 22 package was used for the statistical analysis. The normality of distribution of the data was tested by using the Shapiro–Wilk test. Thereafter, the independent-samples *t*-test was used when the data were normally distributed, while the Mann–Whitney U-test was used when the data were not normally distributed.

## 3. Results

### 3.1. Platelet Count in Liver Transplantation and Control Patients

**Figure 1:**
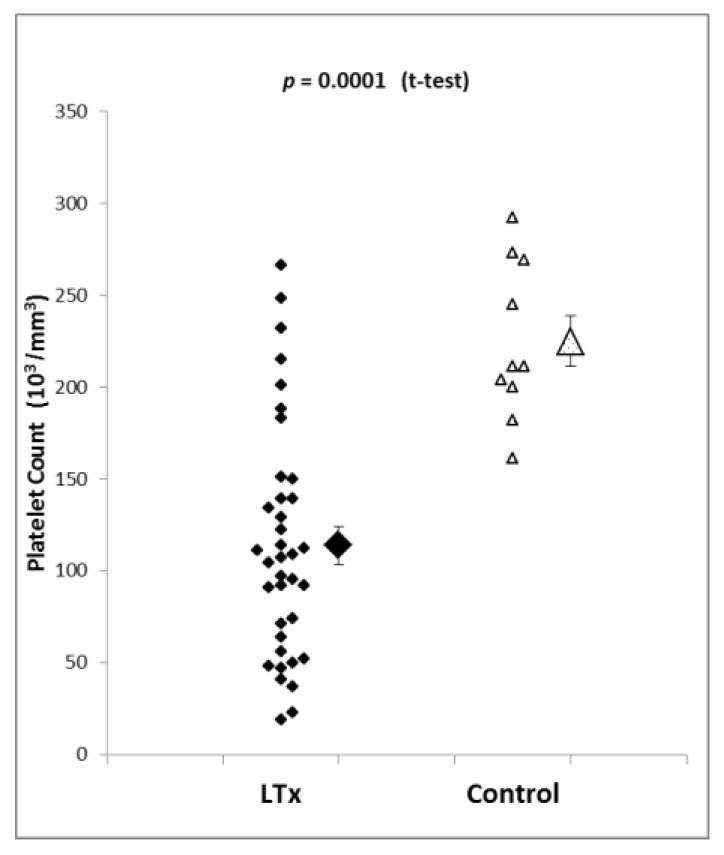
Platelet concentration in the liver transplantation group (LTx, *n* = 37) and control group (*n* = 10). Platelet count is significantly higher in control patients. Shown are both individual measurements and mean and standard error of the mean.

The initial values of platelet count showed a broad distribution in a range from 19 × 10^3^/mm^3^ to 266 × 10^3^/mm^3^ in patients presenting for liver transplantation (*n* = 37) and a comparably narrower distribution in a smaller range of 161 × 10^3^/mm^3^ to 292 × 10^3^/mm^3^ in the control group (*n* = 10) (Figure 1). Statistical evaluation demonstrated significant differences between the liver transplantation and the control group (225 × 10^3^/mm^3^ ± 7 × 10^3^/mm^3^ vs. 114 × 10^3^/mm^3^ ± 20 × 10^3^/mm^3^, mean ± SEM, *p* = 0.0001). During the course of the liver transplantation procedure, platelet count increased from 119 × 10^3^/mm^3^ ± 2 × 10^3^/mm^3^ to 168 × 10^3^/mm^3^ ± 6 × 10^3^/mm^3^ without platelet transfusion (*n* = 31, mean ± SEM, *p* = 0.03), most likely due to the administration of prednisolone (1 g) at the beginning of the transplantation procedure.

### 3.2. Ristocetin-Induced Platelet Aggregation in Liver Transplantation and Control Patients

**Figure 2:**
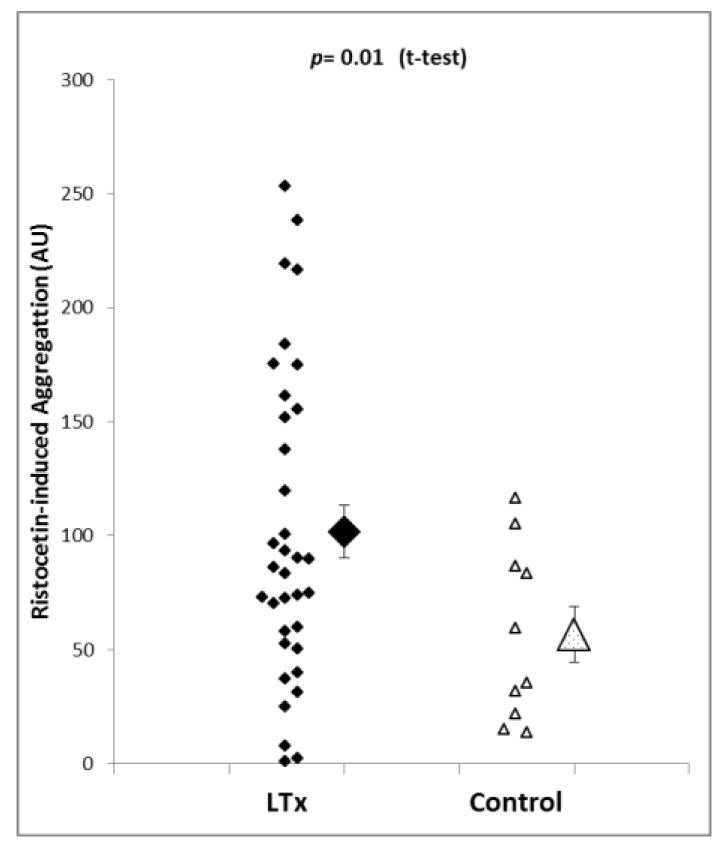
Ristocetin-induced platelet aggregation is significantly higher in liver transplantation patients (*n* = 35) than in the control group (*n* = 10). The results suggest the presence of a highly active von Willebrand factor in liver transplantation patients in comparison to control patients. Shown are individual measurements as well as mean and SEM.

In order to determine von Willebrand factor-induced platelet aggregation, whole-blood samples were activated with ristocetin. Platelet aggregation in the liver transplantation patients using this activator ranged from 1.1 to 253.5 AU, and the mean and SEM were 101.8 ± 11.5 AU. Ristocetin-induced aggregation in the control patients (*n* = 10) was within a range of 13.9 to 116.6 AU, and the mean was 56.9 ± 6.5 AU (mean ± SEM). Statistical analysis revealed that platelet aggregation using the ristocetin test was significantly higher in liver transplantation patients than in control patients (*p* = 0.01, see Figure 2). Importantly, ristocetin-induced aggregation was higher although the platelet count was markedly lower in liver transplantation patients, and the impedance aggregometry results are dependent on platelet count.

**Figure 3:**
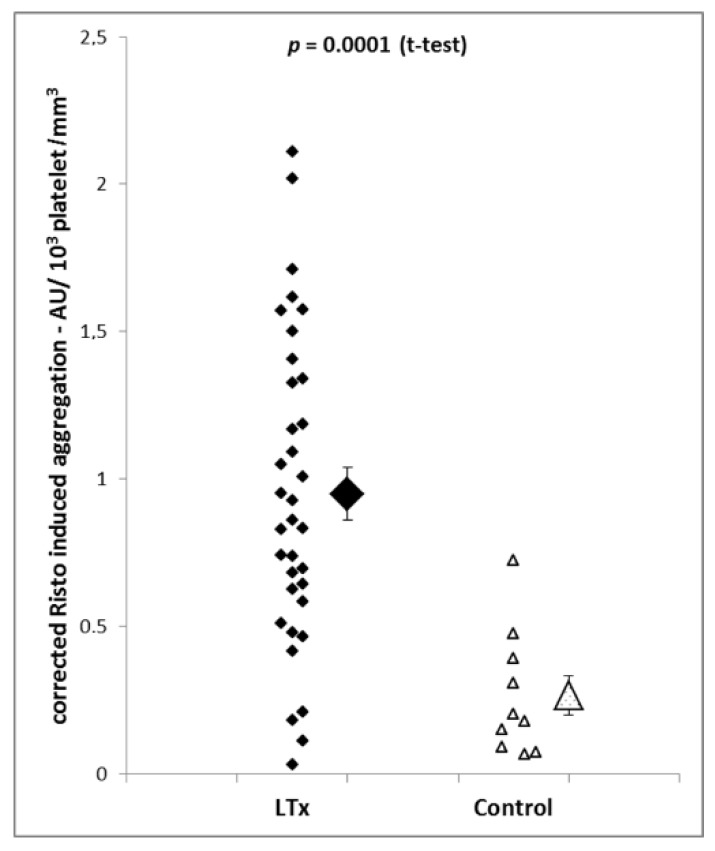
Ristocetin-induced aggregation after correction for platelet count is markedly higher in liver transplantation patients (*n* = 35) than in control patients (*n* = 10). Individual measurements as well as means and SEM of the groups are shown.

To correct the impedance aggregometry findings for platelet count, the ratio between impedance aggregometry readings and platelet count was calculated. The results demonstrated that corrected platelet aggregation was increased 3.1 times in liver transplantation patients (*p =* 0.0001; Figure 3).

**Figure 4:**
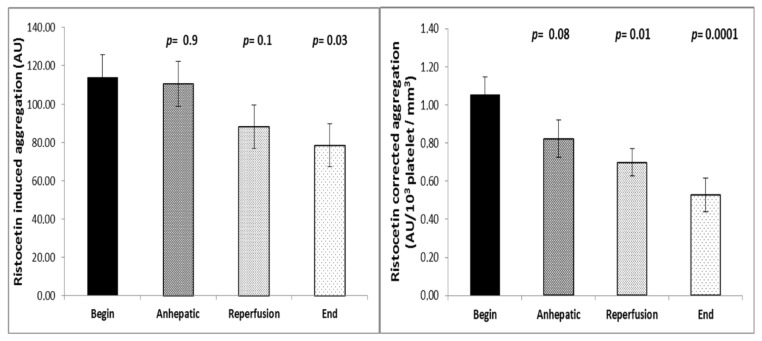
Time course of platelet aggregation during liver transplantation (*n* = 35) using the ristocetin test without and with correction for platelet count. Both corrected and uncorrected platelet function decreases with the reperfusion of the transplant. Values are given as mean and SEM. The Mann–Whitney U-test was used to determine changes in platelet aggregation from the initial value during the course of surgery.

Ristocetin-induced platelet aggregation significantly decreased during the course of surgery. While aggregation was 114.0 ± 2.0 AU at the beginning of the operation, a value of 78.5 ± 2.1 AU was measured at the end of the procedure (mean ± SEM, *n* = 29; *p* = 0.03; Figure 4). When impedance aggregometry findings were corrected for platelet count, the impedance aggregometry ratio decreased from 1.05 AU/10^3^ platelets ± 0.02 AU/10^3^ platelets to 0.53 AU/10^3^ platelets ± 0.02 AU/10^3^ platelets (mean ± SEM; *p =* 0.0001, Figure 4).

### 3.3. ADP-, Arachidonic Acid-, Collagen-, and TRAP-Induced Platelet Aggregation in Liver Transplantation and Control Patients

**Figure 5:**
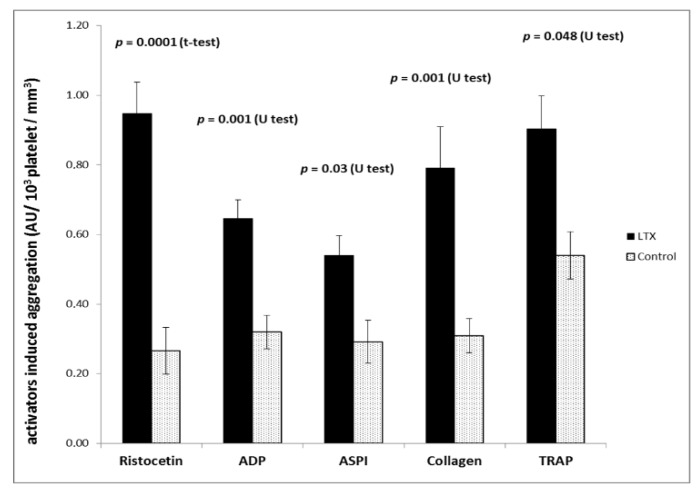
Platelet function ratio (correction for platelet count) in liver transplantation patients (*n* = 35) and control patients (*n* = 10). Platelet aggregation was induced by ristocetin, ADP, arachidonic acid (ASPI), collagen, and thrombim receptor-activating peptide (TRAP) at the begining of the procedure. Data are given as mean and SEM.

Platelet aggregation using ADP, arachidonic acid, collagen, and TRAP was not different in liver transplantation and control patients when the impedance aggregometry readings were not corrected for platelet count. After correction, however, an approximately twofold increase in platelet responsiveness was found in liver transplantation patients. The results of the corrected impedance aggregometry findings are shown in Figure 5.

**Figure 6:**
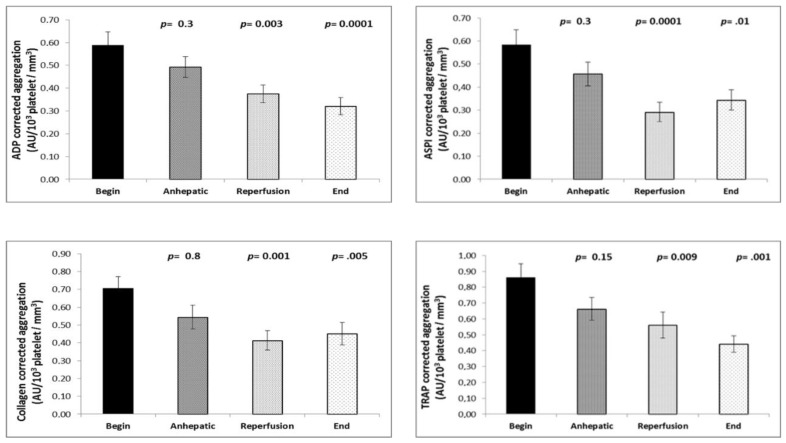
Time course of the platelet aggregation ratio (correction for platelet count) induced by adenosine diphosphate (ADP), arachidonic acid (ASPI), collagen, and thrombin receptor-activating peptide (TRAP) in liver transplantation patients (*n* = 35). Platelet function decreases with the reperfusion of the transplant. Values are given as mean and SEM. The Mann–Whitney U-test was used to determine changes in platelet aggregation from the initial value during the course of surgery.

The determination of platelet aggregation during the course of transplantation revealed a small but significant decrease in platelet aggregation after reperfusion of the liver with all activators without correction for platelet count (ADP-, arachidonic acid-, collagen-, and TRAP). The time course of the aggregation values corrected for platelet count demonstrated an approximately 50% decrease of platelet aggregation with all activators (Figure 6).

## 4. Discussion

The results of the present study demonstrate that platelet aggregation in patients presenting for liver transplantation is markedly increased in comparison to surgical control patients and shows a broad distribution. Platelet aggregation was most pronounced when ristocetin was used as the platelet activator, thus suggesting an increased activity of von Willebrand factor. However, platelet responsiveness was also increased, albeit to a lesser degree, when ADP, arachidonic acid, collagen, or TRAP were used as the activators. In comparison to the start of the liver transplantation procedure, platelet aggregation markedly decreased with the reperfusion of the transplanted liver until the end of surgery. This study is the first to demonstrate that impedance aggregometry as a bedside method is capable to detect increased platelet reactivity in liver transplantation patients. These findings suggest that: 1. The contribution of platelets to hemostasis might not be as compromised as judged by the (reduced) platelet count and varies from patient to patient, 2. Increased platelet reactivity might contribute to the well-known risk of thrombotic complications, and 3. Substitution of platelets based on impedance aggregometry instead of platelet count might be worth being investigated in further studies.

It is well recognized that there are numerous alterations in hemostasis in end-stage liver disease. Components of procoagulant, anticoagulant, and fibrinolytic pathways are reduced, resulting in the so-called rebalanced coagulation system [3]. While, under unstressed conditions, neither bleeding nor thrombosis is observed, the balance of the hemostasis system can easily be disturbed during liver transplantation [14]. During surgery, bleeding can easily lead to dilution coagulopathy. Moreover, hemodynamic shock and tissue damage can result in trauma-induced coagulopathy. Vice versa, several mechanisms including inflammation, tissue factor release, stasis of blood, endothelial activation in the transplant, and blood products can contribute to a procoagulatory state in patients presenting for liver transplantation [1,14].

Platelets have been demonstrated to play an important role in the pathophysiology of hemostasis in end-stage liver disease. Besides bleeding due to thrombocytopenia, thrombosis of hepatic artery or portal vein as well as pulmonary embolism, entrapment of platelets after reperfusion in the transplant, and sinusoidal obstruction syndrome are important pathologies with a potential involvement of platelets [10,11,12,15]. Recently, the involvement of von Willebrand factor/ADAMTS13 has been suggested in thrombotic complications in liver transplantation patients, as well as a role for platelet-induced microthrombus formation in acute liver failure patients [9,16]. Moreover, the transfusion of platelets has been demonstrated to be an independent predictor of liver failure and outcome in liver transplantation patients [17,18,19].

Nowadays, hemostatic management during liver transplantation is often performed with viscoelastic tests (ROTEM™, TEG™), enabling a rapid monitoring of coagulation, platelet count, fibrinolysis, and transfusion of fresh frozen plasma, platelet concentrates, and coagulation factors [20,21]. In contrast, prothrombin time (PT) and activated partial thromboplastin time (aPTT) are thought to be unrelated to the bleeding risk. It is important to state that viscoelastic tests have limitations and are unable to detect alterations in platelet function, and platelet function tests are not routinely used.

Therefore, we used impedance aggregometry with the Multiplate™ device in the present study to determine platelet aggregation using ristocetin, ADP, arachidonic acid, collagen, and TRAP as platelet activators [13]. As the bedside method was performed with whole-blood samples, a measurement of aggregation could be achieved within few minutes during surgery, while classical Born aggregometry is time-consuming and restricted to highly specialized laboratories. Another important difference between these methods is related to the correction for platelet count: In impedance aggregometry, unprocessed whole blood is used, and platelet function readings are not corrected for platelet count, while in Born aggregometry, platelet-rich plasma is diluted to normalize platelet count. This fact must be taken into account for the comparison of impedance aggregometry findings in liver transplantation patients and control surgical patients, as platelet count was about 50% lower in liver transplantation patients. We recently demonstrated a strict proportional relation between platelet count and impedance aggregometry findings in an experimental series with serial dilution of platelet-rich plasma samples (correlation *r* = 0.9). Thus, the impedance aggregometry findings can be corrected for platelet count using the ratio between platelet function and platelet count. This correction provides values which are independent of platelet count and, therefore, are comparable to Born aggregometry values. Both corrected and uncorrected impedance aggregometry findings are of biological significance: While uncorrected values are indicative of the amount of platelets aggregating in response to a certain agonist, corrected values reveal the reactivity of an individual platelet to an agonist.

Impedance aggregometry findings not corrected for platelet count demonstrated an increased aggregation response to ristocetin and unchanged responses to ADP, arachidonic acid, collagen, and TRAP. These results might be indicative of an increased or at least unchanged amount of platelets aggregating at a potential site of lesion. This fact is intriguing in view of the fact that liver transplantation patients’ platelet count was reduced by about 50% in comparison to that of controls. Correction of impedance aggregometry findings for platelet count revealed that the aggregation response of an individual platelet was increased 3.1 times in liver transplantation patients (in comparison to control surgical patients) when ristocetin was used as the activator, and was increased twofold when ADP, arachidonic acid, collagen, and TRAP were used. While the increased ristocetin-induced aggregation can be attributed to increased von Willebrand factor levels in end-stage liver disease, the cause for the increased response to the other agonists is unknown.

The time course of platelet aggregation showed a decline of agonist-induced aggregation at the end of surgery. The decrease in ristocetin-induced platelet aggregation is in good agreement with laboratory evaluations demonstrating that the capacity of von Willebrand factor to react with platelets normalized at the end of surgery [15]. Thus, the risk of platelet transfusion might be lower at the end of surgery.

The present study has limitations. The number of patients was low, and their diseases were heterogeneous. Further large studies are necessary to investigate whether intraoperative impedance aggregometry findings can predict bleeding, thrombosis, transplant function, or mortality and whether interventions might improve the prognosis of patients. However, the present study is the first to show that impedance aggregometry reveals highly variable and increased platelet aggregation during liver transplantation.

## Abbreviations

ADP	Adenosine triphosphate
AU	Arbitrary units
TRAP	Thrombin receptor-binding peptide
